# Disseminated septic arthritis caused by *Ureaplasma urealyticum* in an immunocompromised patient with hypogammaglobulinemia after rituximab therapy

**DOI:** 10.1007/s15010-024-02301-1

**Published:** 2024-06-10

**Authors:** Kohei Oguni, Shinnosuke Fukushima, Yuki Otsuka, Yoshiaki Soejima, Marina Kawaguchi, Yosuke Sazumi, Takumi Fujimori, Koji Iio, Noriyuki Umakoshi, Kazuki Yamada, Hideharu Hagiya, Fumio Otsuka

**Affiliations:** 1 Department of General Medicine, Graduate School of Medicine, Dentistry and Pharmaceutical Sciences, Okayama University, 2-5-1 Shikata-cho, Kitaku, Okayama, 700-8558 Japan; 2 Department of Infectious Diseases, Okayama University Hospital, Okayama, Japan; 3 Department of Bacteriology, Graduate School of Medicine, Dentistry and Pharmaceutical Sciences, Okayama University, Okayama, Japan; 4 Microbiology Division, Clinical Laboratory, Okayama University Hospital, Okayama, Japan; 5 Department of Radiology, Okayama University Hospital, Okayama, Japan; 6 Department of Medical Materials for Musculoskeletal Reconstruction, Faculty of Medicine, Dentistry and Pharmaceutical Sciences, Okayama University, Okayama, Japan

**Keywords:** *Ureaplasma urealyticum*, Joint infection, Rituximab, Hypogammaglobulinemia, α-Defensin, 16S rRNA gene sequencing

## Abstract

**Purpose:**

*Ureaplasma urealyticum* is a rare pathogen associated with septic arthritis that predominantly affects patients with hypogammaglobulinemia. Bacterial identification of fastidious organisms is challenging because they are undetectable by routine culture testing. To the best of our knowledge, this is the first report of septic arthritis induced by *U. urealyticum* infection in Japan.

**Case description:**

We describe the case of a 23-year-old Japanese female with secondary hypogammaglobulinemia (serum immunoglobulin level < 500 mg/dL), identified 8 years after treatment with rituximab. The patient presented with persistent fever and polyarthritis that were unresponsive to ceftriaxone and prednisolone. Contrast-enhanced computed tomography and gallium-67 scintigraphy revealed effusion and inflammation in the left sternoclavicular, hip, wrist, knee, and ankle joints. Although Gram staining and bacterial culture of the drainage fluid from the left hip joint were negative, the condition exhibited characteristics of purulent bacterial infection. The patient underwent empirical treatment with doxycycline, and her symptoms promptly resolved. Subsequent 16S ribosomal RNA (rRNA) gene sequencing of the joint fluid confirmed the presence of *U. urealyticum,* leading to the diagnosis of septic arthritis*.* Combination therapy with doxycycline and azithromycin yielded a favorable recovery from the inflammatory status and severe arthritic pain.

**Conclusion:**

This case highlights *U. urealyticum* as a potential causative agent of disseminated septic arthritis, particularly in patients with hypogammaglobulinaemia. The 16S rRNA gene analysis proved beneficial for identifying pathogens in culture-negative specimens, such as synovial fluid, in suspected bacterial infections.

## Introduction

Members of the family *Mycoplasmataceae,* including *Ureaplasma and Mycoplasma* species, colonize the human genitourinary tract. *Ureaplasma* spp. can be isolated from cervical and vaginal secretions, ranging from 31.2 to 72.1% among fertile females and from 72.3 to 80% among pregnant females [1, 2], possibly causing non-gonococcal urethritis, prostatitis, gynecological diseases, and infertility [3, 4]. The diagnosis of *Ureaplasma* infections without clinical suspicion is challenging because these organisms are untraceable by both Gram staining and bacterial culture [3]. *Ureaplasma* species may also cause extragenital and disseminated infections in immunocompromised patients, such as those with hypogammaglobulinemia [5], hematologic malignancies [6], and solid organ transplantation [7]. Septic arthritis caused by *Ureaplasma* spp., although rare, has been documented in patients with hypogammaglobulinemia [5]. Rituximab, an anti-CD20 chimeric monoclonal antibody, is associated with secondary hypogammaglobulinemia [8]. A recent case of *U. urealyticum* septic arthritis has been reported in a patient who developed hypogammaglobulinemia after rituximab therapy [5]. Although the diagnosis is difficult, 16S ribosomal RNA (rRNA) sequencing is used to detect *Ureaplasma* species [9]. PCR testing for 16S rRNA is an effective method for identifying pathogens that are undetectable by routine microbiological testing [10, 11]. Nevertheless, 16S rRNA gene analysis is not commonly used in Japan, and its clinical use remains limited. To the best of our knowledge, no case of *Ureaplasma* septic arthritis has been reported in Japan.

Herein, we present a case of septic arthritis caused by *U. urealyticum*, successfully diagnosed using 16S rRNA gene sequencing in a patient previously treated with rituximab for nephrotic syndrome.

## Case report

A 23-year-old Japanese female was referred to our department for fever and polyarthritis. Approximately 8 years prior to this presentation, the patient had undergone four cycles of rituximab for glucocorticoid-resistant nephrotic syndrome. Two months prior, she had been hospitalized for empyema and underwent surgical debridement; however, the pathological and microbiological etiologies remained unclear. Subsequently, she developed fever and polyarthritis affecting the elbow, shoulder, hip, wrist, and ankle joints. At the previous hospital, ceftriaxone and 30 mg/day of prednisolone were initiated for suspected septic and reactive arthritis; however, her symptoms did not improve, and she became bedridden because of severe joint pain.

Upon admission, she was alert and her vital signs were stable, except for fever. Physical examination revealed severe tenderness of the left wrist, left sternoclavicular joint, left ankle joint, and both hip joints. Laboratory testing demonstrated an elevation in white blood cell count (20,920/µL) and serum C-reactive protein (CRP, 23.36 mg/dL). Serum levels of IgG, IgA, and IgM decreased to 243.3 mg/dL (normal range, 861.0–1747 mg/dL), 20.4 mg/dL (normal range, 93.0–393.0 mg/dL), and < 2.0 mg/dL (normal range, 50.0–269.0 mg/dL), respectively, leading to a diagnosis of hypogammaglobulinemia, for which we promptly initiated immunoglobulin replacement therapy. Both antinuclear and antineutrophil cytoplasmic antibodies tested negative. Immunoglobulin levels were confirmed to be within normal limits before receiving rituximab.

**Fig. 1:**
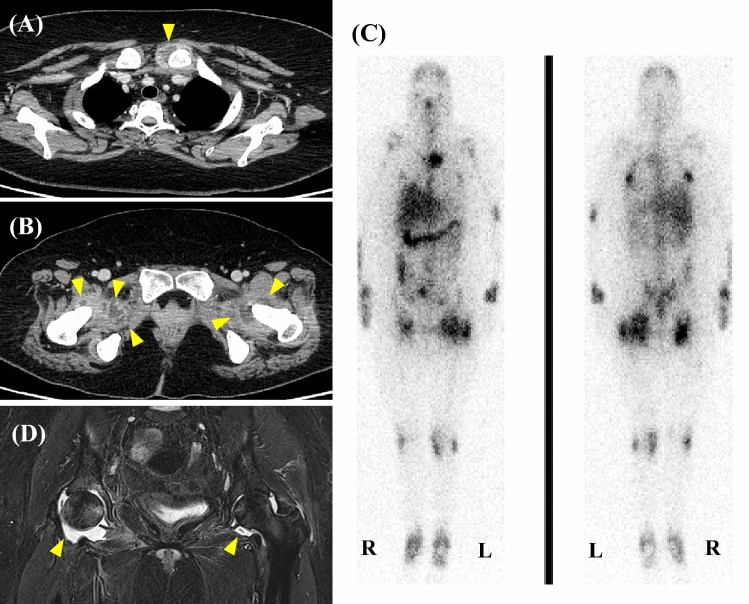
Radiological findings **A**,** B** Contrast-enhanced computed tomography showing effusions in the left sternoclavicular and both hip joints. **C** Gallium-67 scintigraphy demonstrating uptakes in bilateral wrist, knee, ankle joints, and large joints. **D** Magnetic resonance imaging of the hips detecting bone marrow edema along with fluid retention bilaterally

Contrast-enhanced computed tomography (CT) revealed effusions in the left sternoclavicular and bilateral hip joints (Fig. 1A, B). Gallium-67 scintigraphy revealed uptake in the left sternoclavicular, hip, wrist, knee, and ankle joints (Fig. 1C). CT-guided drainage of the left hip joint detected yellowish, cloudy joint fluid, with an increased cell count (60,000/µL) showing a predominance of polynuclear cells (99%) over mononuclear cells (1%). Despite these findings, Gram staining and bacterial culture of the joint fluid did not reveal any pathogens. While, we observed a positive testing result for alpha-defensin lateral flow tests (Synovasure^®^ lateral flow test; Zimmer Biomet, IN, USA) in synovial fluid of both hips.

**Fig. 2:**
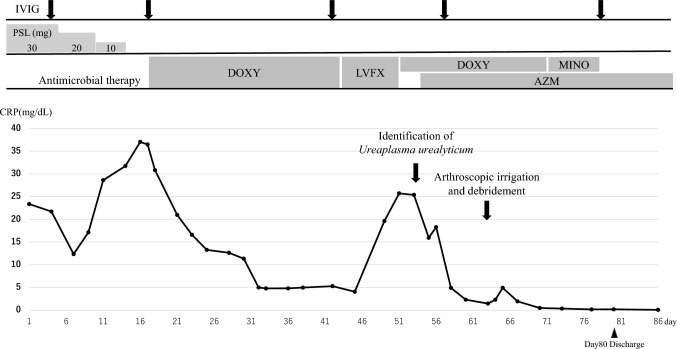
The clinical course of the patient Serum C-reactive protein levels decreased following DOXY therapy and resurged after switching to LVFX. Combination therapy with DOXY (MINO) and AZM was effective, and the patient was discharged with AZM monotherapy. *PSL* prednisolone; *CRP* C-reactive protein; *IVIG* intravenous immunoglobulin; *DOXY* doxycycline;* MINO* minocycline; *LVFX* levofloxacin; *AZM* azithromycin

Suspecting septic arthritis caused by atypical organisms, treatment with 200 mg doxycycline (DOXY) daily was initiated. Following antibiotic therapy, the joint symptoms and serum C-reactive protein (CRP) levels improved (Fig. 2). Although clinically effective, DOXY therapy was switched to 500 mg/day levofloxacin (LVFX) because of DOXY-induced gastritis, as confirmed using gastroendoscopy. Soon after, the serum CRP level was elevated during LVFX therapy, accompanied by severe joint tenderness, suggesting LVFX resistance. We then switched to oral DOXY therapy. Gastric symptoms recurred, and DOXY was converted to intravenous minocycline.

Around that time, we performed an *in-house* two-step PCR for the 16S rRNA gene to accurately identify the causative organism. DNA was isolated from hip joint fluid using the DNeasy® PowerSoil Pro Kit (QIAGEN). The 16S rRNA gene was amplified with 8UA and 1485B primers (forward primer: 5′-AGAGTTTGATCMTGGCTCAG-3′; reverse primer: 5′-TACGGTTACCTTGTTACGAC-3′). PCR was performed using the following regimen: 98 °C for 3 min followed by 45 cycles at 98 °C for 4 s, 60 °C for 30 s, 72 °C for 1 min, and a final extension at 72 °C for 5 min (ramp rate = 1 °C/s). The second PCR was carried out with 341A and 519B primers (forward primer: 5′-CTACGGGAGGCAGCAGTGGG-3′, and reverse primer: 5′-ATTACCGCGGCKGCTG-3′) under a following amplification process: 96 °C for 1 min, followed by 25 cycles at 96 °C for 10 s, 50 °C for 5 s, 60 °C for 1 min (ramp rate = 1 °C/s). The sequence data of the PCR products were analyzed using the Basic Local Alignment Search Tool (BLAST), and the isolate was identified as *U. urealyticum* with a 100% concordance rate with the reference strain (GenBank accession number: NR_041710.1).

Under the diagnosis of disseminated arthritis caused by *U. urealyticum,* we added oral azithromycin (500 mg for 3 days, followed by 250 mg/day) as combination therapy to prevent the emergence of antimicrobial resistance. Magnetic resonance imaging revealed bilateral joint effusions, bone marrow edema in both hip joints (Fig. 1D) and fluid retention in the ankle joints. Thereafter, arthroscopic irrigation and debridement of both hip joints were performed 63 days after admission. During hospitalization, the patient remained bedridden because of severe joint tenderness, even after intravenous fentanyl therapy. Combination therapy with antibiotics and surgical intervention led to an improvement in her fever and polyarthritis. Intravenous minocycline was discontinued and the patient was discharged in good condition on oral azithromycin, with plans to continue antibiotic treatment for several months.

## Discussion

*Ureaplasma* joint infection is a rare entity, predominantly reported in patients with hypogammaglobulinemia [5, 9, 12, 13]. Although patients with *Ureaplasma* joint infections often present with preceding urogenital infections, there were no prior infectious episodes in this case. Instead, the patient experienced an empyema episode of unknown etiology. *Ureaplasma* rarely causes respiratory infections [14], and it remains uncertain whether the fastidious pathogen was responsible for the empyema observed in the present case.

*Ureaplasma* species are difficult to detect using routine assays, and PCR testing is often used to identify these organisms [15]. Although PCR testing for *Ureaplasma* species is commercially available for urine specimens in Japan, no equivalent commercial tests exist for other types of specimens such as joint fluids. The absence of testing options complicates the diagnosis of joint infections caused by *Ureaplasma* species. In this case, the alpha-defensin, a synovial fluid biomarker used for diagnosing periprosthetic joint infection, tested positive, having supported our presumptive diagnosis of bacterial infection [16]. Sequencing of the 16 s rRNA gene, which is ubiquitously present in bacteria, is a critical tool for identifying difficult-to-culture organisms [10]. There have been several reports on *Ureaplasma* detection using 16S rRNA gene analysis [15, 17, 18]. Given that only a limited number of medical facilities can perform the 16 s rRNA gene analysis, it is crucial to collect and preserve specimens from the infection site before initiating empiric therapy.

*Ureaplasma* species are generally susceptible to antibiotics such as tetracyclines, fluoroquinolones, and macrolides [13]. In a study of 48 clinical isolates of *U. urealyticum* from the United States, all isolates were susceptible to tetracyclines and macrolides, whereas resistance to LVFX was observed in 5.2% of the cases [19]. In contrast, in China, the resistance rate of *U. urealyticum* to LVFX ranges between 60 and 82% [2, 4], indicating significant geographical variation in resistance patterns. In our case, the clinical deterioration following the switch from DOXY to LVFX implied the presence of an LVFX-resistant strain. The optimal treatment duration for joint infections caused by *Ureaplasma* remains unknown. Nevertheless, we considered that an extended treatment period of several months may have been preferable in the present case, as evidenced by the relapse in a patient following a 6-week treatment regimen [13].

Hypogammaglobulinemia can be classified into primary and secondary types. Most secondary hypogammaglobulinemia cases are associated with B cell-targeted therapies, such as rituximab [20]. The incidence of rituximab-associated hypogammaglobulinemia requiring immunoglobulin replacement therapy has been reported to be 6.6% in patients with lymphoma and 14–21% in those with ANCA-associated vasculitis [21]. The median interval between initial rituximab administration and onset of hypogammaglobulinemia was 18 months, and the prevalence of hypogammaglobulinemia reportedly ranged from 13–17% during an observation period of up to 60 months [22]. In the present case, the patient’s immunoglobulin levels were within the normal range before rituximab administration, suggesting a low likelihood of primary hypogammaglobulinemia. However, the onset of hypogammaglobulinemia was not determined because immunoglobulin levels were not monitored after rituximab administration. Severe hypogammaglobulinemia was diagnosed in this patient 8 years after the last rituximab administration. It is advisable to examine immunoglobulin levels after B cell-targeted therapy and administer immunoglobulin replacement therapy as needed [21].

Here, we present a case of *U. urealyticum* disseminated arthritis in an immunocompromised patient with hypogammaglobulinemia. We highlighted that 16S rRNA gene analysis is a valuable tool for the diagnosis of culture-negative infections.

## Data Availability

No datasets were generated or analysed during the current study
